# Nitrosamines crisis in pharmaceuticals − Insights on toxicological implications, root causes and risk assessment: A systematic review

**DOI:** 10.1016/j.jpha.2023.12.009

**Published:** 2023-12-12

**Authors:** Hemanth P.R. Vikram, Tegginamath Pramod Kumar, Gunjan Kumar, Narasimha M. Beeraka, Rajashree Deka, Sheik Mohammed Suhail, Sandeep Jat, Namitha Bannimath, Gayatiri Padmanabhan, Ravandur S. Chandan, Pramod Kumar, Bannimath Gurupadayya

**Affiliations:** a Department of Pharmaceutical Chemistry, JSS College of Pharmacy Mysuru, JSS Academy of Higher Education and Research (JSSAHER), Mysuru, 570015, India; b Xenone Healthcare Pvt. Ltd., New Delhi, 110076, India; c Department of Pharmaceutics, JSS College of Pharmacy Mysuru, JSS Academy of Higher Education and Research (JSSAHER), Mysuru, 570015, India; d Department of Human Anatomy, I.M. Sechenov First Moscow State Medical University (Sechenov University), Moscow, 119991, Russian Federation; e Department of Pharmacology, Raghavendra Institute of Pharmaceutical Education and Research (RIPER), Ananthapuramu, 515721, India; f Herman B. Wells Center for Pediatric Research, Department of Pediatrics, Indiana University School of Medicine, Indianapolis, IN, 46202, USA; g Animal Physiology and Biochemistry Laboratory, Department of Zoology, Gauhati University, Guwahati, 781014, India; h Department of Pharmacology, JSS College of Pharmacy Mysuru, JSS Academy of Higher Education and Research (JSSAHER), Mysuru, 570015, India; i Department of Pharmaceutical Analysis, National Institute of Pharmaceutical Education and Research (NIPER)-Guwahati, Changsari, 781101, India; j Department of Pharmacology, University of Galway, Galway, H91 TK33, Ireland

**Keywords:** Carcinogenicity, Genotoxicity, Mutagenicity, Molecular toxicity, Nitrosamine impurities, Nitrosamine drug substance-related impurities

## Abstract

The presence of *N*-nitroso compounds, particularly *N*-nitrosamines, in pharmaceutical products has raised global safety concerns due to their significant genotoxic and mutagenic effects. This systematic review investigates their toxicity in active pharmaceutical ingredients (APIs), drug products, and pharmaceutical excipients, along with novel analytical strategies for detection, root cause analysis, reformulation strategies, and regulatory guidelines for nitrosamines. This review emphasizes the molecular toxicity of *N*-nitroso compounds, focusing on genotoxic, mutagenic, carcinogenic, and other physiological effects. Additionally, it addresses the ongoing nitrosamine crisis, the development of nitrosamine-free products, and the importance of sensitive detection methods and precise risk evaluation. This comprehensive overview will aid molecular biologists, analytical scientists, formulation scientists in research and development sector, and researchers involved in management of nitrosamine-induced toxicity and promoting safer pharmaceutical products.

## Highlights

• The nitrosamines crisis in pharmaceuticals is discussed.
• We delve into the toxicological implications and toxicity profiles of *N*-nitroso compounds.
• We discussed root cause analysis, risk assessment, and mitigation strategies for nitrosamine impurities.
• We highlighted GC and LC methods for detection and quantification of *N*-nitroso compounds in pharmaceuticals.
• The emphasis is on developing nitrosamine-free pharmaceutical products to ensure regulatory compliance.

## Graphical abstract

**Figure:**
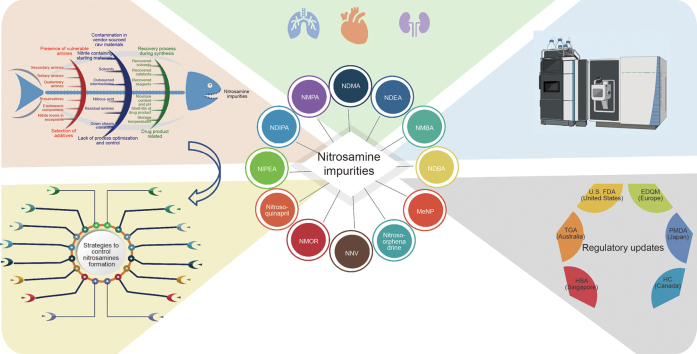

## 1 Introduction

Nitrosamine impurities, even in trace amounts, are highly toxic and mutagenic, capable of damaging DNA, and subsequently increase the risk of cancer incidence [1]. Regulatory authorities have identified nitrosamine impurities in various active pharmaceutical ingredients (APIs) and other approved medications leading to the abrupt recall of drugs like sartans (valsartan), ranitidine (zantac), nizatidine, metformin, and varenicline due to unacceptable levels of nitrosamine impurities [2].

In recent times, regulatory bodies have shifted their focus to a newer class of nitrosamine impurities known as nitrosamine drug substance-related impurities (NDSRIs), which share structural similarity with the API. Recent incidents involving NDSRIs include quinapril hydrochloride where the presence of *N*-nitroso-quinapril was detected [3]. Orphenadrine citrate enteric release tablets which exhibited *N*-nitroso orphenadrine as an impurity [3]. *N*-nitroso-varenicline (NNV) in varenicline is formed through a reacting between drug substance and nitrites present in the excipients [4].

**Fig. 1:**
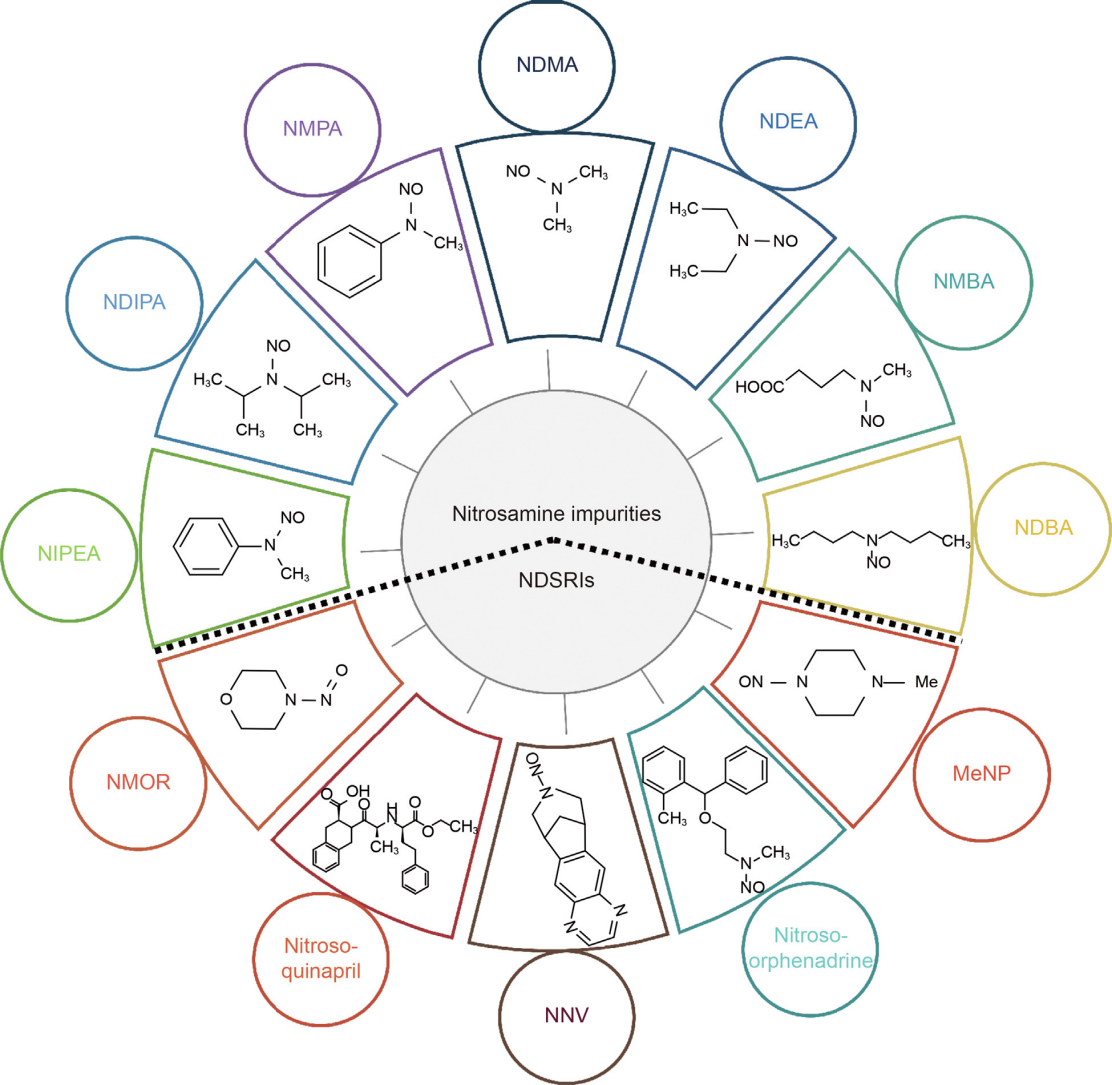
Chemical structures of various nitrosamine impurities and nitrosamine drug substance-related impurities (NDSRIs). NDMA: *N*-nitrosodimethylamine; NDEA: *N*-nitrosodiethylamine; NMBA: *N*-nitroso-*N*-methyl-4-aminobutanoic acid; NDBA: *N*-nitrosodibutylamine; MeNP: 1-methyl-4-nitrosopiperazine; NNV: *N*-nitroso-varenicline; NMOR: *N*-nitrosomorpholine; NIPEA: *N*-nitrosoisopropylethyl amine; NDIPA: *N*-nitrosodiisopropylamine; NMPA: *N*-nitrosomethylphenylamine.

Nitrosamine impurities are classified as Class 1 known mutagenic impurities according to The International Council for Harmonisation of Technical Requirements for Pharmaceuticals for Human Use (ICH) M7 guidelines [5]. The International Agency for Research on Cancer (IARC) categorized these impurities into Groups 2A and 2B based on epidemiological data [6] with *N*-nitrosodimethylamine (NDMA) and NDEA falling under Group 2A, while other nitrosamines are classified as Group 2B human carcinogens [7]. The chemical structures of selected nitrosamines and NDSRIs are represented in Fig. 1.

To address the nitrosamine crisis, the United States Pharmacopeia (USP) is hosting a virtual platform called the “nitrosamines exchange”. This platform brings together pharmaceutical companies, contract research organizations (CROs), API manufacturers, and excipients suppliers to facilitate real-time communication, updates, and risk assessments related to the nitrosamines issue. Additionally, USP has established a “nitrosamine analytical hub” to provide updates on novel analytical methods for testing nitrosamines.

**Fig. 2:**
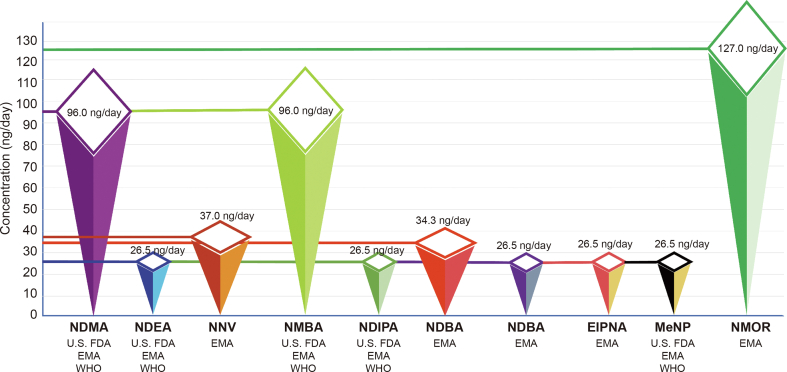
Acceptable daily intake limits for selected nitrosamines impurities and nitrosamine drug substance-related impurities (NDSRI) in medicinal products as per regulatory authorities. NDMA: *N*-nitrosodimethylamine; U.S. FDA: United States food and drug administration; EMA: European medicines agency; WHO: World Health Organization; NDEA: *N*-nitrosodiethylamine; NNV: *N*-nitroso-varenicline; NMBA: *N*-nitroso-*N*-methyl-4-aminobutanoic acid; NDIPA: *N*-nitrosodiisopropylamine; NMPA: *N*-nitrosomethylphenylamine; NDBA: *N*-nitrosodibutylamine; EIPNA: *N*-nitrosoethylisopropylamine; MeNP: 1-methyl-4-nitrosopiperazine; NMOR: *N*-nitrosomorpholine.

Regulatory authorities for drug control have specified acceptable daily intake limits for various nitrosamine impurities and NDSRIs in APIs and medicinal products. For instance these limits include 96 ng/day for *N*-nitrosodimethylamine (NMDA) and *N*-nitroso-*N-*methyl-4-aminobutanoic acid (NMBA), 26.5 ng/day for *N*-nitrosomethylphenylamine (NMPA), *N*-nitrosodiisopropylamine (NDIPA), *N*-nitrosoisopropylethyl amine (NIPEA), *N*-nitrosodiethylamine (NDEA), and 1-methyl-4-nitrosopiperazine (MeNP), 34.3 ng/day for NMPA, 37 ng/day for NNV, and 127 ng/day for *N*-nitrosomorpholine (NMOR) [5,8,9]. These limits are represented in Fig. 2.

**Fig. 3:**
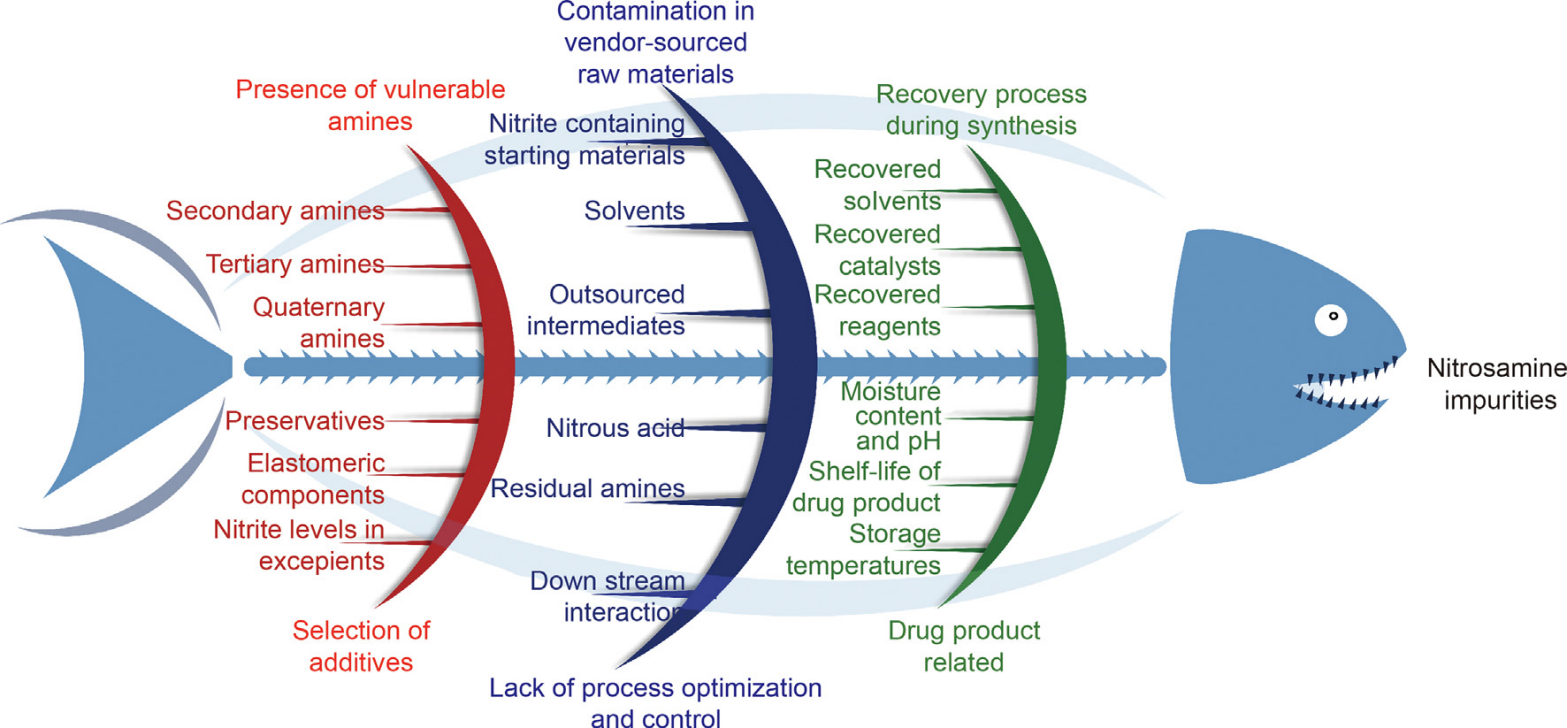
Root causes and sources of nitrosamine impurities in active pharmaceutical ingredients (APIs) and medicinal products.

Conditions that fosters nitrosamine formation include the presence of vulnerable amines, such as secondary and tertiary amines [10]. These amines react with nitrous acid in acidic conditions, leading to the formation of nitrosamines. Nitrous acid itself is unstable and is formed from nitrites (NO_2_^−^) under acidic conditions. Nitrosamine impurities may also originate from contamination in vendor-sourced raw materials, including nitrite-containing starting materials, intermediates, and the reuse of solvents, reactants, catalysts and reagents during synthesis. During the manufacturing of drug products, several factors, such as the selection of additives, preservatives, excipients, and elastomeric components, should be carefully assessed, as they can contribute to the generation of nitrosamines in pharmaceutical products. Additionally, post-production factors such as moisture content, pH, and storage temperatures of the product might also lead to generation of nitrosamines [11]. Formulators of APIs or drug products should consider these factors and take necessary steps to mitigate nitrosamine impurities in pharmaceutical products. Moreover, nitrosamines may also form in the gastrointestinal tract if vulnerable amines, along with nitrites, are consumed [12]. The root causes and sources of nitrosamine impurities in pharmaceutical products are represented in the form of a fishbone diagram in Fig. 3.

The recall of drugs due to the nitrosamine crisis has resulted in disruptions in supply chains and shortage of several drugs in markets, raising concerns among patients globally. It has also impacted the clinical drug development process. Drug interaction studies were affected by the recall of key drugs such as metformin, rifampin, ranitidine, nizatidine, and propranolol. Experimental studies and *in silico* approaches could be handy to theoretically predict the formation of nitrosamines in numerous drugs containing secondary and tertiary amines.

This systematic review significantly examines and discusses the toxicity of *N*-nitroso compounds, primarily focusing on *N*-nitrosamines, in drug products, pharmaceutical excipients, and APIs. Additionally, it encompasses recent updates on the molecular toxicity profiles of these impurities within physiological systems. Furthermore, the review investigates novel, updated analytical strategies for the sensitive detection and quantification of these hazardous impurities. It delves into the root causes for the presence of nitrosamine impurities and offers insightful reformulation strategies for pharmaceutical products. Additionally, it provides regulatory updates aimed at mitigating the occurrence of these nitrosamine impurities.

## 2 Literature search

We conducted a literature search by accessing public databases, including PubMed, Medline, eMedicine, the National Library of Medicine (NLM), and ReleMed. We focused on published articles, including original research, review articles, case reports, and other systematic reviews, both with and without comprehensive meta-analysis, related to *N*-nitroso compounds in pharmaceutical excipients, APIs, and drug products. Subsequently, we collected information relevant to the updated molecular toxicity implications associated with nitrosamine impurities and the implications of multilevel approaches for sensitive detection. Our primary screening of 180 articles yielded the relevant data for this study. All data gathered for this study adhered to the guidelines outlined in the Preferred Reporting Items for Systematic reviews and Meta-Analyses (PRISMA) statement. We conducted an automatic search with manual sorting of the selected articles.

### 2.1 Study selection

**Scheme 1:**
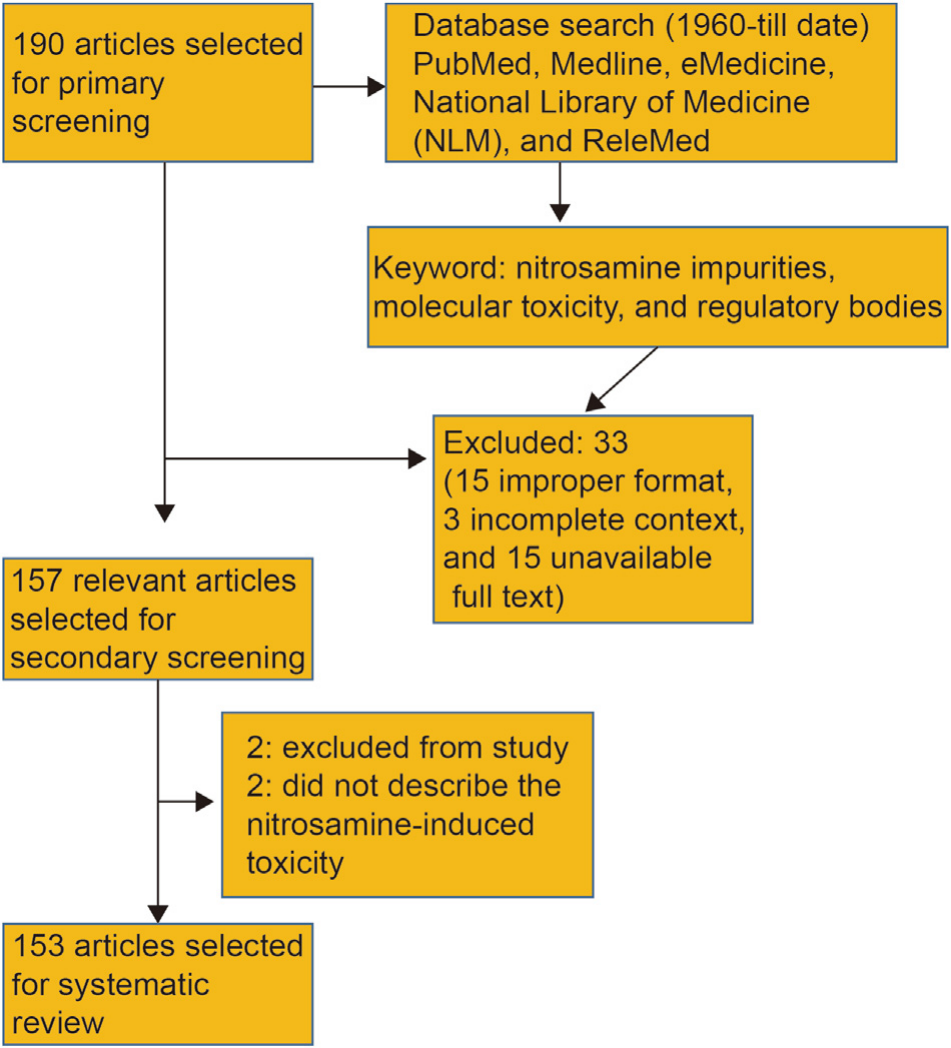
The schematic depiction of the exclusion and inclusion criteria for articles included in the study through primary and secondary screening.

For this systematic review, all published or accepted articles pertaining to nitrosamine-related topics, specifically focusing on toxicity and analytical implications in pharmaceuticals, were included. A total of 180 articles spanning from 1960 to the present day, including original research, reviews, case reports and studies reporting nitrosamine impurities above the no-observed-adverse-effect levels (NOAEL) established by regulatory agencies, were initially screened. During the primary screening, we considered factors such as relevance, publication date, access to the full article text, and content. Subsequently, in the secondary screening phase, we excluded the 33 reports including 15 with improper formats (such as case reports, letters, and short communications), 3 with incomplete content, and 15 with unavailable full texts. This process resulted in the selection of 147 articles for further evaluation. Of these, 2 articles were excluded from the study and 2 articles did not describe nitrosamine-induced toxicity or its pharmaceutical or analytical implications. In the end, 143 articles were chosen for systematic review. The schematic depiction of the exclusion and inclusion criteria for articles included in the study through primary and secondary screening is represented as Scheme 1.

Our initial focus was to gather information on nitrosamine-induced toxicity, side effects, or physiological toxicity triggered by nitrosamine impurities. Additionally, we aimed to explore analytical techniques for sensitive detection of nitrosamine impurities and to put forth regulatory updates on nitrosamine impurities.

### 2.2 Data extraction

In accordance with international standards outlined in the “Systematic review of observational studies in epidemiology”, data selection and categorization followed specific criteria. These criteria included the number of articles addressing nitrosamine-induced toxicity, other neurological or systemic complications, year and type of publication, and the preclinical studies describing signaling pathways implicated in nitrosamine-induced toxicity for toxicity management. We organized the data into three main groups to facilitate categorization: articles that describe “side effects or physiological toxicity triggered by nitrosamine impurities”, those focusing on “analytical techniques for sensitive detection of nitrosamine impurities”, and those aiming to “put forth regulatory updates on nitrosamine impurities”. The results of these three categories were reviewed and analyzed separately. This systematic approach allowed us to address the crucial inferences, which may prove valuable to molecular biologists, analytical scientists, formulation scientists in research and development sector, and researchers involved in management of nitrosamine-induced toxicity.

## 3 Molecular toxicity of nitrosamine impurities

NDMA, NDEA, *N*-nitrosoethylisopropylamine (NEIPA), NDIPA, *N*-nitrosodibutylamine (NDBA), and NMBA are the predominant nitrosamines that can foster the carcinogenic effects via genotoxicity or mutagenicity in the physiological system. In recent years, it has become evident that impurities in several pharmaceutical products pose significant human health risks, as observed in rodent models. For example, NMBA and NDEA have metabolic similarities to the NMDA and 12 carcinogenic *N*-nitrosomethyl-*N-*alkylamines (NMAs). Both genotoxicity and tumorigenicity are assessed through the examination of chromosomal aberrations, sister chromatid exchange, and micronuclei formation during studies on nitrosamines-induced toxicity studies.

### 3.1 Genotoxicity

Nitrosamine impurities such as NDMA, NDEA, and others can induce mutagenic effects and subsequently thereby leading to carcinogenicity. NDMA has been shown to induce mutations due to the genotoxicity *in vivo* models [[13], [14], [15], [16], [17], [18]]. Cytochrome P450 (CYP450) enzymes such as CYP2E1 and CYP2A6 are involved in the metabolism of *N*-nitrosamines and form *N*-nitrosodialkylamines [[19], [20], [21]]. For instance, in rodent models, CYP2E1 is implicated in the metabolism of several substrates including organic solvents, nitrosamines, and other drug molecules [22]. Additionally, CYP3A4 plays a role in catalyzing the activation of larger *N*-nitrosamines [23]. The differential expression of CYP2E1 modulates the nitrosamines metabolism in different *in vitro* or *in vivo* models [21,24,25].

Genotoxicity of 4-(methylnitrosamino)-1-(3-pyridyl)-1-butanone (NNK)-mediated DNA damage includes the formation of methyl DNA base adducts, such as *O*^6^-Me-dGuo and *N7*-Me-dGuo. Other genotoxic products resulting from the activity of *N*-nitrosamines in the healthy cells include methyl DNA base adducts and pyridyloxobutyl (POB) DNA base adducts including *O*^6^-POB-dGuo, and *O*^2^-POB-Thd. Aldehyde DNA adducts are also significant genotoxic products formed due to the presence of nitrosamine impurities. However, pyridylhydroxybutyl DNA adducts require further extensive studies to explore the exact nature of nitrosamine impurities [1,26,27]. Table 1 [7,[28], [29], [30], [31], [32], [33], [34], [35], [36], [37], [38], [39], [40], [41], [42], [43], [44], [45], [46], [47], [48], [49], [50], [51], [52], [53], [54], [55]] outlines the carcinogenic potential of *N*-nitroso compounds across various routes of administration in several *in vivo* models.

**Table 1:** The carcinogenic potential of *N*-nitroso compounds across various routes of administration in several *in vivo* models.

*N*-nitroso compounds	No.	Route of administration	Animal models	Tumors	Refs.
*N*-nitrosodimethylamine (NMDA)	1	Oral	Mice, rats, hamsters, rabbits, guinea pigs, and fish	Benign and malignant tumors of the liver	[7,28]
	2	Inhalation	Mice, prenatal exposure in mice	Benign and malignant tumors of the liver	[7,28]
	3	Subcutaneous	Hamsters, mastomys, and newborn and suckling mice and rats	Benign and malignant tumors of the liver	[7,28]
	4	Intraperitoneal	Adult and newborn mice and in newts	Benign and malignant tumors of the liver	[7,28]
	5	Intramuscular	Rats	Benign and malignant tumors of the liver	[7,28]
	6	Oral	Frogs and fish	Benign and malignant tumors of the liver	[7,28]
	7	Oral	Lung tumors in mice	Tumors of the respiratory tract	[7,28]
	8	Inhalation	Mice and rats	Lung tumors	[7,28]
	9	Inhalation	Rats	Nasal-cavity tumors	[7,28]
	10	Subcutaneous	Adult, newborn, and suckling mice and nasal cavity tumors in adult hamsters	Lung tumors	[7,28]
	11	Intraperitoneal	Adult and newborn mice	Lung tumors	[7,28]
	12	Inhalation	Rats	Nasal-cavity tumors	[7,28]
	13	Inhalation	Mice	Lung tumors	[7,28]
	14	Oral exposure	Mice, rats, and hamsters	Blood-vessel tumors	[7,28]
	15	Subcutaneous	Hamsters and adult, newborn, and suckling mice	Blood-vessel tumors	[7,28]
	16	Intraperitoneal	Mice	Blood-vessel tumors	[7,28]
	17	Oral	Frogs	Tumors of the hematopoietic system	[7,28]
	18	Oral	Mollusks	Tumors of the digestive gland and hematopoietic system	[29]
	19	Subcutaneous	Female hamsters	Ovarian tumors	[30]
	20	Oral	Foxes	Liver tumors	[31]
	21	Intraperitoneal	Rats	Lung and liver tumors	[32]
	22	Oral	Humans	Oropharyngeal cancer	[33]
	23	Oral		Stomach	[34,35]
	24	Oral		Esophageal	[[36], [37], [38], [39], [40]]
	25	Oral		Colorectal	[41]
*N*-nitrosodiethylamine (NDEA)	1	Inhalation	Rats	Liver tumors	[7,28]
	2	Rectal	Rats	Liver tumors	[7,28]
	3	Intramuscular	Chickens and cats	Liver tumors	[42]
	4	Intraperitoneal	Newborn mice	Liver tumors	[43,44]
	5	Intratracheal	Hamsters of both sexes	Tumors of the lung or trachea	[[45], [46], [47]]
	6	Subcutaneous	Rabbits	Tumors of the lung or trachea	[48]
	7	Intraperitoneal	Newborn mice	Tumors of the lung or trachea	[44]
	8	Oral	Larval or juvenile fish	Pancreatic tumors	[49]
	9	Oral	Mollusks	Tumors of the digestive gland and hematopoietic system	[29]
	10	Intraperitoneal	Pregnant hamsters, prenatally exposed offspring, and second generation of offspring	Benign laryngotracheal tumors	[50]
*N*-nitrosomorpholine (NMOR)	1	Oral	Male hamsters, female rats, and male hamsters	Respiratory or digestive-tract tumors	[51]
	2	Intravesicular	Rats	Tumors of internal organs	[52]
	3	Oral	Female rats	Tumors of the esophagus	[7]
	4	Oral	Male mice	Hepatocellular adenoma	[7]
	5	Oral	Rats	Hepatocellular carcinoma and cholangiofibroma	[7]
	6	Intravenous	Rats	Liver cancer	[7]
	7	Oral	Syrian golden hamsters	Malignant liver tumors	[[53], [54], [55]]
	8	Oral	Hamsters	Respiratory or digestive-tract tumors	[55]
	9	Oral	Hamsters	Tumors of the respiratory tract and upper digestive tract	[55]

Furthermore, DNA lesions are formed due to the activity of these nitrosamines in pharmaceutical ingredients or drugs, leading to the formation of *O*^6^-methylguanine or 3-methyladenine (3MeA) [1,[56], [57], [58], [59]]. DNA methylation is primarily induced by NDMA, and the unrepaired 3MeA can promote mutagenicity, subsequently leading to cancer. Typically, the DNA damage induced by 3MeA is repaired by the alkyladenine glycosylase, which eliminates the methylated bases, triggering base excision repair [1]. Therefore, the activity of alkyladenine glycosylase is crucial in minimizing the mutagenicity induced by nitrosamine impurities such as NDMA [[60], [61], [62]].

### 3.2 Carcinogenicity

Several epidemiological reports have described the influence of nitrosamines on the risk of developing esophageal cancers, colon cancers, hepatocellular cancers, and other devastating forms of cancers [[63], [64], [65]].

#### 3.2.1 Lung cancer

*Lung cancer* can be induced by the nitrosamine impurities, as they have the ability to stimulate proliferation, migration, and cancer cell survival by modulating G-protein signaling, nicotinic acetylcholine receptor (nAchR) signaling, and epidermal growth factor receptor (EGFR) signaling. For instance, nitrosamines' influence on EGFR signaling can induce changes in the cyclic adenosine monophosphate-protein kinase A-rapidly accelerated fibrosarcoma-mitogen-activated protein kinase kinase-extracellular signal-regulated kinase 1/2 (cAMP-PKA-RAF-MEK-ERK1/2) axis, subsequently promoting cancer cell proliferation. Similarly, the EGFR modulation could cause alterations in the phosphoinositide-3-kinase-Akt-mammalian target of rapamycin (PI3K-Akt-Mtor) signaling pathway to fostering cancer cell survival. Nitrosamines also induce changes in nAChR signaling, voltage gated calcium channel (VGCC), calcium signaling, and protein kinase C (PKC) modulation, which contribute to cancer progression through migration and survival. HIF1-alpha signaling is also modulated by the beta-adrenergic signaling when nitrosamines interact with these receptors which subsequently cause angiogenesis for nutrient supply to the proliferating cancer cells.

#### 3.2.2 Esophageal cancer

Esophageal cancer has higher incidence rates and wide geographical prevalence, indicating the involvement of environmental factors in its development. Past reports in toxicogenomics have described the role of nitrosamines in esophageal carcinogenesis. A study by Zhao et al. [64] elucidated the potential of nitrosamines to induce carcinogenicity in the esophageal epithelium. In this study, the authors examined the urinary levels of nitrosamines in patients associated with esophageal squamous cell carcinoma (ESCC), basal cell hyperplasia, and precancerous lesions, comparing them to healthy individuals. The risk was found to be higher in ESCC patients with elevated levels of NMEA, NDBA, nitrosopyrrolidine (NPYR), and NMOR compared to the control group. As per this report, there is a higher risk of ESCC in patients with hazardous exposure to *N*-nitrosomethylethylamine (NMEA), NDBA, and NPYR, suggesting a specific dose-response relationship between nitrosamine exposure and the incidence of esophageal cancer [64].

#### 3.2.3 Hepatocellular and pancreatic cancer

Hepatocellular cancer is one of the lethal cancers that can result from the metabolism of nitrosamines with the involvement of P450 enzymes. Lifestyle factors, such as dietary exposure to higher levels of nitrosamines or *N*-nitroso compounds, have been linked to the development of cancer [66,67]. These compounds specifically form DNA adducts in the nuclei of cells located in digestive organs and the liver contributing to the carcinogenicity of nitroso compounds [68,69]. Furthermore, food processing additives containing these compounds serve as a significant source of nitrosamine exposure in humans [70,71]. A study by Zheng et al. [65] explored the relationship between the dietary intake of specific *N*-nitroso compounds, total *N*-nitroso compounds, and the development of hepatocellular carcinoma. The authors concluded that higher consumption of *N*-nitroso compounds could increase the risk of hepatocellular carcinoma incidence, in conjunction with other risk factors such as hepatitis B virus (HBV) or hepatitis C virus (HCV) infection or alcohol intake.

**Fig. 4:**
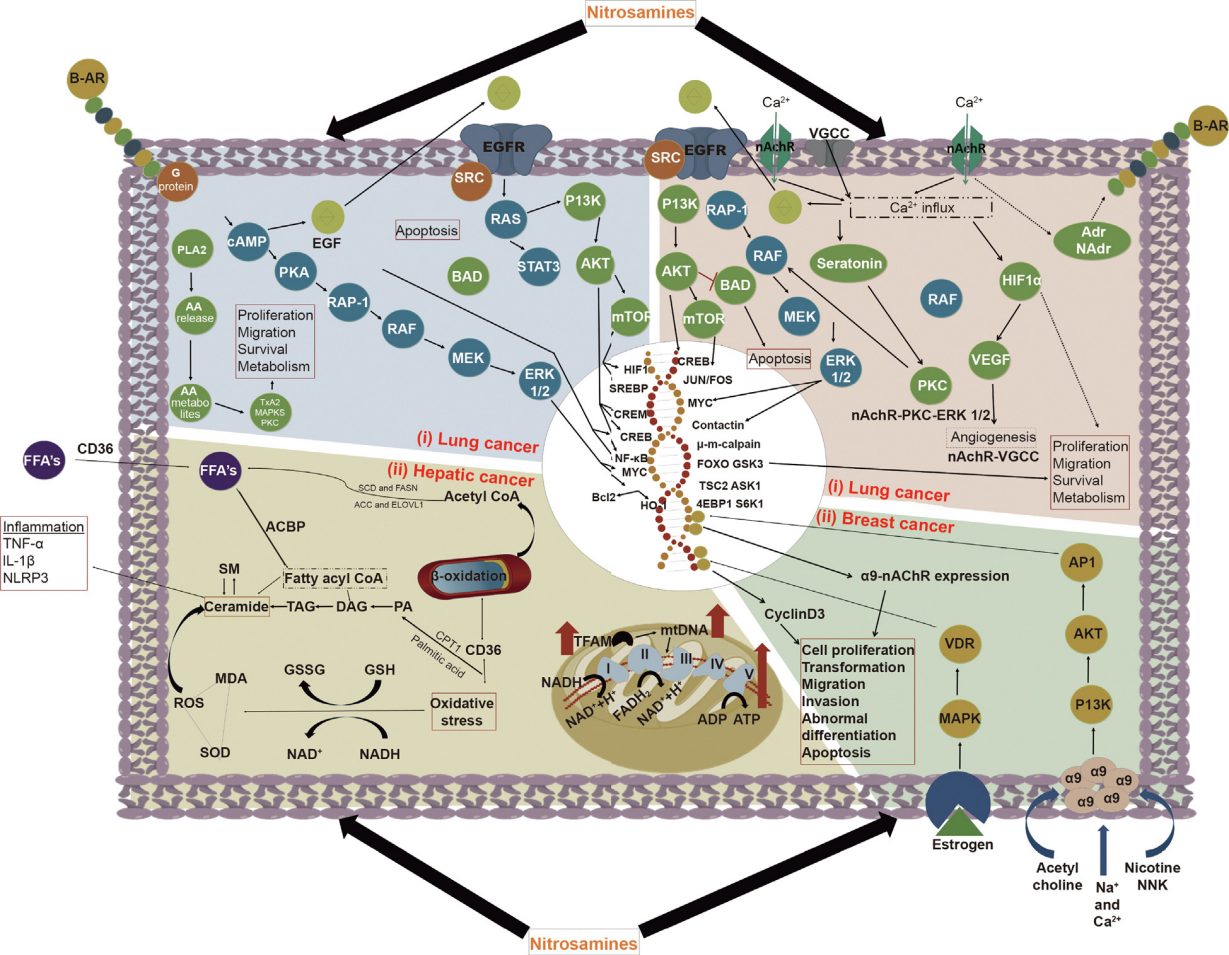
Molecular signaling pathway for nitrosamines in various types of cancers. (i) Lung cancer: nitrosamines can stimulate proliferation, migration and cancer cell survival by modulating G-protein signaling, nAchR signaling, and EGFR signaling. (ii) Breast cancer: nitrosamines modulates adenylyl cyclase/cAMP/PKA/CREB pathway, which mediates EGFR signaling pathway transactivation. (iii) Hepatic cancer: *N*-nitrosamines exposure affects hepatic lipid metabolism through the alteration of mitochondrial DNA, OXPHOS signaling, and other mitochondrial metabolic pathways. I: nicotinamide adenine dinucleotide (NADH)-ubiquinone oxidoreductase complex; II: succinic acid-ubiquinone oxidoreductase complex; III: cytochrome Bc1 complex; IV: cytochrome C oxidase complex; and V: adenosine triphosphate synthase complex; B-AR: β-adrenergic receptor; Adr: adrenaline; NAdr: noradrenaline; HIF1α: hypoxia inducible factor 1α; VEGF: protein kinase B; RAF: rapidly accelerated fibrosarcoma; PKC: protein kinase C; ERK: extracellular signal-regulated kinase; MEK: mitogen‑activated protein kinase kinase; RAP-1: Ras-related protein 1; BAD:  BCL2 associated agonist of cell death protein mTor: mammalian target of rapamycin; PI3K: phosphoinositide 3-kinase; VGCC: voltage gated calcium channel; nAchR: nicotinic acetylcholine receptors; CREB: cAMP response element-binding protein; FOS, JUN and Myc: transcription factors; μ-m calpain: isozymes of calpain; FOXO: forkhead transcription factor; GSK3: glycogen synthase kinase 3; TSC2: tuberous sclerosis 2; ASK1: apoptosis signal-regulating kinase 1; 4EBP1: eukaryotic translation initiation factor 4E (eIF4E)-binding protein 1; S6K1: S6 kinase 1; PLA2: phospholipase-A2; PKA: protein kinase A; TxA2: thromboxane A2; SREBP-1: sterol regulatory element-binding protein 1; CREM: cAMP Response Element Modulating protein; NF-kB: nuclear factor kappa B; Bcl2: B cell lymphoma 2; HO-1: heme oxygenase-1; AA: arachidonic acid; RAS: rat sarcoma proteins; STAT: signal transducer and activator of transcription; AP1: activator protein 1; PI3K: phosphoinositide 3-kinase; MAPK: mitogen-activated protein kinase; VDR: vitamin D receptor; FASN: fatty acid synthase; ROS: reactive oxygen species; CD36: fatty acid translocase; SOD: superoxide dismutase; ACC: acetyl-CoA carboxylase; TNF-α: tumor necrosis factor alpha; IL-1β: interleukin-1β; SCD: stearoyl-CoA desaturase; ELOVL1: ELOVL fatty acid elongase 1; ACBP: acyl-CoA binding protein, TAG: triacylglycerols; DAG: diacylglycerols; PA: phosphatidic acids; CPT1: carnitine palmitoyltransferase 1; GSH: glutathione; GSSG: glutathione disulfide; MDA: malondialdehyde; NAD: nicotinamide adenine dinucleotide; TFAM: mitochondrial transcription factor-A.

Mainly, NDEA and NDMA are significant nitroso compounds found in pharmaceuticals and these are classified as Group 2A carcinogens for humans. Metabolic activation by CYP450 enzymes can induce electrophilic alkylation, leading to the formation of mutation-induced DNA adducts [68]. Hepatic enzymes play a substantial role in the metabolism of nitrosamines, with greater activity observed in the liver when compared to the other extrahepatic tissues [72]. Dose-response relationships concerning nitrosamines have shown that NDMA and NDEA induce hepatic tumors in *in vivo* models. DNA adduct formation has been observed in both *in vivo* models and human in liver tumors with significant nitrosamines exposure [[72], [73], [74], [75], [76], [77]]. *N*-nitrosamines induce the hydroxylation of an alpha-carbon atom within the alkyl group, generating hydroxyl nitrosamines in liver cells and subsequently fostering DNA alkylation. These sequential events further cause gene damage, mutations, and confers malignant transformation of liver cells into cancer cells by triggering oncogenic signaling [[78], [79], [80], [81]] as represented in Fig. 4. Furthermore, exposure to *N*-nitrosamines affects hepatic lipid metabolism through alterations in mitochondrial DNA, oxidative phosphorylation (OXPHOS) signaling, and other mitochondrial metabolic pathways. Another mechanism of nitrosamines involves the induction of inflammation in liver tissue through oxidative stress, adenosine triphosphate (ATP) depletion, and a decline in enzymes related to fatty acid oxidation [82].

NDEA and NDMA are considered as the pancreatic carcinogens as indicated by the positive correlation for the intake of certain foods that can induce generation of *N*-nitroso compounds [71]. This study also investigated the dietary intake of factors such as cigar smoking, vitamin C, vitamin E, or red meat in relation to the incidence of pancreatic cancer. Notably, the intake of vitamins C and E could be considered as nitrosation inhibitors while the consumption of heme iron in red meat may contribute to the endogenous production of *N*-nitroso compounds [70,[83], [84], [85], [86]].

Pentoxifylline has demonstrated the potential to ameliorate hepatotoxicity and oxidative stress induced by *N*-nitrosamines in experimental rodent models [87]. The *N*-nitrosamines role in damaging mitochondrial lipid metabolism yet requires substantial studies to investigate the exact toxicology of these impurities in pharmaceuticals [82,[88], [89], [90]].

#### 3.2.4 Breast cancer

Breast cancer remains a major cause of mortality and morbidity worldwide, often attributed to unhealthy lifestyle patterns and dietary habits. Tobacco contains a wide range of nitrosamines, with NNK being a significant carcinogen known to promote the malignant cancer cells [91]. These tobacco derived carcinogens are particularly relevant to the development of various cancers including breast cancer. NNK plays a significant role in the development and progression of breast cancer by interacting with the nAChR present in cancer cells. Notably, the α9nAChR receptor is implicated in the development and progression of breast cancer cells [92,93]. Additionally, nicotine and NNK can interact with this receptor system, subsequently influencing cancer cell proliferation by modulating crucial cell signaling pathways like mitogen-activated protein kinase (MAPK) and PI3K-Akt pathways. These signaling pathways are stimulated by the activity of nicotine and estrogen hormone stimulation. The activation of α9nAChR by NNK and nicotine forms a significant feedback loop through PI3K-Akt and MAPK signaling, which could further initiate activator protein 1 (AP1) and vitamin D receptor (VDR) transcription factors stimulation to confer to the cancer development [[94], [95], [96], [97]]. This phenomenon has been confirmed through gene knockdown studies of α9nAChR using small interfering RNA (siRNAs) [[98], [99], [100]]. However, the current nitrosamine impurities found in pharmaceutical formulations and APIs are yet to be investigated for their potential to modulate the activity of α9nAChR in fostering breast cancer through *in vitro* and *in vivo* studies.

Moreover, the beta-adrenergic receptor system, part of the G-protein coupled receptor system, and its downstream signaling play a significant role in promoting cancer cell proliferation upon NNK activity [101,102]. This signaling pathway subsequently fosters the modulation of the adenylyl cyclase/cAMP/PKA/cAMP response element-binding protein (CREB) pathway, which, in turn, mediates the transactivation of EGFR signaling pathway [103,104]. However, the specific impact of nitrosamine impurities on the modulation of these G-protein coupled signaling pathways and EGFR pathways is yet to be investigated vividly through *in vitro* and *in vivo* studies for their potential role in incidence of cancers.

### 3.3 Physiological alterations: nitrosamine impurities in angiotensin receptor blockers

NDMA, NDEA, NEIPA, NDIPA, NDBA, and NMBA are major nitrosamines known to induce carcinogenicity in the physiological system [105]. Additionally, these impurities found in pharmaceutical drugs could cause alterations in the physiological system. For instance, angiotensin receptor blockers (ARBs) such as losartan, valsartan, olmesartan, irbesartan, and telmisartan have been recalled from US markets due to elevated levels of nitrosamine impurities in their products, which can disrupt physiological homeostasis [105]. The presence of these nitrosamines in ARBs is primarily attributed to improper manufacturing process [106].

NMDA is metabolized by the P450 2E1 in hepatic microsomes in humans [20]. Methyl group oxidation could induce the formation of to α-hydroxy-NDMA and this is mutagenic which can simultaneously decompose and generate two reactive species including formaldehyde and methyl diazohydroxide [20,107]. These two metabolites may have significant effect on the physiological systems [108]. In case of NDEA, the metabolic activation that cause oncogenesis is profoundly mediated by the P450 2E1 and P450 2A6 [20,109,110]. Ethyl diazohydroxide is formed from the P-450 mediated hydroxylation of NDEA, which further forms ethyldiazonium that can interact with DNA and induce formation of DNA adducts. The formation of DNA could cause the significant alteration in the physiology of healthy cells in human tissues and transform into neoplastic cell by the oncogenicity. Rodent studies pertinent to the effects of NDEA described the incidence of several kinds of liver cancers, esophageal cancers, and nasopharyngeal cancers, because toxic metabolite induced physiological alterations and oncogenicity [16,111].

DNA adducts such as *O*^6^-methylguanine (*O*^6^-Me-Gua) and *N*7-Me-Gua induced by the NDMA when incubated with esophagus cell cultures obtained from human tissues [112]. Methylated DNA adducts observed across the human tissues could typically damage the healthy cells [[113], [114], [115]]. In case of liver cell DNA, the NDMA-poisoned victims, *O*^6^-Me-Gua and *N*7-Me-Gua formation was observed. In case of NMEA, *N7*-Me-Gua is methylated form of DNA adducts formed substantially in hepatic region and renal region followed by the esophagus and lungs. These toxic DNA adducts may further harm the cell protein formation at transcriptional and translational levels which required substantial future studies to unravel the underlying mechanisms behind these *N*-nitroso compounds-induced physiological alterations [116].

## 4 Analytical techniques to enhance sensitive detection of nitrosamine impurities

For many years, nitrosamine impurities have been reported in pharmaceutical products. However, there are still numerous products containing these impurities that have not been thoroughly explored yet. The recent surge in the withdrawals of pharmaceutical products from markets has raised significant concerns among regulatory bodies worldwide. As a response, regulatory authorities like United States Food and Drug Administration (U.S. FDA), European Medicines Agency (EMA), World Health Organization (WHO) are urging all the concerned stakeholders to perform stringent identification, reporting of these impurities, and are recommending APIs and drug product manufacturers to develop highly sensitive analytical methods, especially gas and liquid chromatographic methods capable of quantifying nitrosamines with lower limit of quantitation (LOQ) levels. This is essential for the effective detection and quantification of trace levels of these impurities in raw materials, intermediates in drug synthesis, and finished drug products, ultimately ensuring the safety of pharmaceuticals for consumers. The development of such novel and sensitive methods is a challenging task that demands substantial expertise and research experience. Major challenges in development of analytical methods can arise from issues such as inadequate ionization of analytes and interference of matrices. When it comes to NDSRIs, lower molecular weights, complexity in the structure, and lack of readily available standards can pose difficulties in achieving sensitive detection. It also necessitates access to high-cost instrumentation, including liquid chromatography tandem mass spectrometry (LC-MS/MS) and gas chromatography-tandem mass spectrometry (GC-MS/MS).

LC-MS/MS and GC-MS techniques are widely employed in nitrosamine detection as they offer good sensitivity with nanograms level detection of nitrosamines. Orbitrap and quadrupole time of flight (Q-TOF) analyzers are preferable for trace-level detection of these impurities. USP's initiative “Nitrosamines Exchange” provides access to industry experts, researchers, and drug manufacturers for the latest advancements in chromatographic techniques for nitrosamine detection. A sensitive analytical method should be capable of quantifying specific nitrosamine impurities in certain matrices (APIs or drug products) to 10% of its acceptable intake limit for that particular nitrosamine impurity [117].

GC-MS/MS is a preferable analytical tool for the identification and quantification of volatile and semi-volatile analytes. Chemical ionization offers better specificity and sensitivity for the analysis of nitrosamine impurities than electron impact ionization [118]. The stationary phases used in GC-MS/MS for nitrosamine analysis include polyethylene glycol (PEG)-coated capillary columns [[119], [120], [121], [122], [123]], cyanopropylphenyl dimethylpolysiloxane [124], and phenyl methylpolysiloxane [125]. Several detectors employed for nitrosamine analysis include the flame ionization detector [125], nitrogen phosphorous detector (NPD) [[126], [127], [128], [129], [130]], and chemiluminescence detector [[127], [128], [129]]. GC-MS/MS analysis can have potential interference with residual solvents, which could affect the robustness and sensitivity of the analytical method [118], for instance, dimethylformamide (DMF) interference in the analysis of NDMA in metformin [131,132]. Interferences from complex matrices can be overcome by additional clean-up and pre-treatment steps like solid-phase extraction (SPE) [124] or liquid-liquid extraction (LLE) [126,133], and the usage of internal standards [121]. Controlling and mitigating the coelution of matrices is essential as it can lead to false positive results, shorten the life span of GC columns, and contaminate the mass spectrometer [134]. This issue can be overcome by using head space (HS) injection mode [[120], [121], [122]]. However, the HS injection mode is not suitable for the analysis of thermolabile analytes like metformin and ranitidine [120,122,125].

Other alternative approaches employed in GC-MS/MS analysis include solvent-free-HS-GC/MS [122] and full-evaporation headspace-GC/MS, which offers higher sensitivity than HS-GC/MS [132]. The burden of procuring costly isotopic internal standards can be overcome by using approaches such as HS-GC/MS [120], HS-solid-phase microextraction (SPME)-GC/MS [121], dispersive liquid-liquid microextraction (DLLME)-GC/MS [125], and full evaporation static headspace gas chromatography method with nitrogen phosphorous detection [132] which do not require the usage of internal standards.

LC-MS/MS is a preferable analytical tool for the identification and quantification of polar, nonpolar, and non-volatile analytes [135]. Lee et al. [136] stated that atmospheric pressure chemical ionization (APCI) has higher sensitivity for nitrosamine analysis compared to electro spray ionization (ESI). ESI is the preferred choice for the analysis of *N*-nitrosodipropylamine (NDPA) [137,138]. Interference from the matrix can be minimized by using atmospheric pressure photo ionization (APPI) [136]. The positive mode of ionization is suitable for most nitrosamines, whereas the negative mode is preferred in the case of NMBA [[139], [140], [141]]. The U.S. FDA suggests the usage of LC-MS/MS for the analysis of metformin and ranitidine due to their thermally unstable nature [[120], [121], [122],^125^]. Deuterated isotopic standards are commonly used in LC-MS/MS analysis. Sample preparation steps are also involved in LC-MS/MS analysis. LLE is more cost effective than SPE [134]. Dichloromethane is the preferred extraction solvent chosen for NDMA analysis [[142], [143], [144]]. Matrix interference in LC-MS/MS can be overcome by an online diverting valve, which bypasses matrices present in the sample [134].

In this systematic review, we have diligently summarized the latest developments in analytical methods, specifically gas and liquid chromatographic methods, for testing nitrosamines in APIs and drug products. This comprehensive summary aims to provide valuable insights to researchers interested in developing more sensitive methods for nitrosamine detection in various drugs. Table S1 [121,124,[139], [140], [141],[145], [146], [147], [148], [149], [150], [151], [152], [153], [154], [155], [156], [157], [158], [159], [160], [161], [162], [163], [164], [165], [166], [167], [168], [169], [170], [171], [172], [173], [174], [175]] presents LC-MS/MS, GC-MS/MS, and HPLC methods that have been recommended by regulatory bodies, independent researchers, and industry experts for nitrosamine analysis.

## 5 Regulatory updates on nitrosamine impurities

In mid-2018, the initial detection of nitrosamines began with NDMA in sartan APIs, which prompted the recall of batches from the markets. International regulatory authorities, such as U.S. FDA, EMA, European Directorate for the Quality of Medicines & Healthcare (EDQM), Health Sciences Authority (HAS, Singapore), Pharmaceuticals and Medical Devices Agency (PMDA, Japan), Therapeutic Goods Administration (TGA, Australia), and Health Canada (HC, Canada), collaborated to alert API manufacturers. They put forth several regulations, recommendations, and guidances to review their manufacturing process and to initiate risk assessments regarding nitrosamines formation [[176], [177], [178]]. The primary focus was on evaluating raw materials, intermediates, solvents, and reagents used in API manufacturing for the presence of vulnerable amines [176]. In 2018, valsartan products were recalled from the market. In 2019, ranitidine, nizatidine, and metformin extended-release products were found to contain nitrosamines, leading to their recall from the market in 2020. The root cause of elevated nitrosamines levels in ranitidine products was linked to storage conditions [179]. Subsequently, attention shifted onto examining key factors that might cause the formation of nitrosamines in drug products.

Regulatory bodies have intended the manufacturers to perform root cause analysis to determine how nitrosamines are incorporated into drugs and drug products. The U.S. FDA put forth a three-step mitigation strategy to control and prevent nitrosamine formation in APIs and drug products. Step 1, manufacturers should prioritize risk assessments for their API and drug product portfolios, and they must document and report their findings to regulatory bodies by March 31, 2021 (U.S. FDA). EMA gave a timeline of March 31, 2021 (chemical medicines), and till July 1, 2021 (biological medicines). Step 2, confirmatory testing using sensitive analytical techniques to quantify nitrosamines is necessary until October 1, 2023 (U.S. FDA), September 26, 2022 for chemical medicines, and July 1, 2023 for biological medicines by EMA. Step 3, manufacturers must report the identified root causes of nitrosamine formation and implement of changes to their processes or approaches to eliminate nitrosamine formation [176,180].

In 2020, Lhasa Limited (UK) initiated the creation of a database on nitrite levels in excipients used in drug product manufacturing. This initiative involved representation from industry experts and researchers, as nitrite levels in excipients make drug products more prone to nitrosamine impurities [181]. Additionally, in 2020, the Committee for Medicinal Products for Human Use (CHMP), constituted by EMA, conducted scientific review of the entire situation and submitted its report in this regard. In 2021, the European medicines regulatory network established an intellectual group known as “Nitrosamine Implementation Oversight Group” to discuss developments and the current scenario regarding the emergence of nitrosamine impurities. EMA asked marketing authorization holders of sartans, rifampicin, ranitidine, metformin-containing, and varenicline medicines to release their products into the markets only after stringent testing [180].

The U.S. FDA primarily recommended following steps 2 and 3 of the three-step mitigation strategies in response to the emergence of NDSRI issues. They also called upon all concerned stakeholders to review, discuss, provide information, and update their suggestions and approaches for regulatory bodies to effectively address this problem. The recent findings on nitrosamines in several products and the subsequent recalls represent just the tip of the iceberg. There is a looming possibility of a higher incidence and risk associated with numerous products already in the market in the coming days. To address this potential crisis, a united task force should be formed in collaboration with all global regulatory bodies with an aim of devising a strategy to tackle the upcoming crisis.

## 6 Nitrosamine risk assessment and reformulation strategies to mitigate incorporation of nitrosamine impurities into pharmaceutical products

Root causes analysis on sources of nitrosamines incorporation must be conducted if nitrosamines are reported in the initial analysis. Subsequently, reformulation strategies must be designed to minimize the risk of nitrosamine impurities formation in pharmaceutical products. Research on reformulation strategies to mitigate nitrosamines in drug products shall be highly beneficial to tackle the crisis caused by nitrosamines.

U.S. FDA recommends the inclusion of antioxidants like ascorbic acid and α-tocopherol, as they have shown the potential to inhibit the generation of nitrosamines within the human body [182,183]. Additionally, the usage of excipients like sodium carbonate, which create a basic pH environment *in vivo*, could be an effective mitigation strategy because the generation of nitrosamines occurs mostly in an acidic medium [182]. Such reformulation strategies can be designed and suggested to the formulators across various pharmaceutical industries and also communicated to regulatory authorities through supplements or amendments submitted by the formulators.

Nitrosamines can be incorporated into drug products in multiple routes, during the API multi-step synthesis process, as well as drug product storage and transport. In context of API synthesis, drugs containing vulnerable amines are particularly prone to nitrosamine contamination [184]. In such cases, API manufacturers should adopt a meticulous approach when selecting catalysts, reagents, and solvents for the multi-step synthesis process [184].

**Fig. 5:**
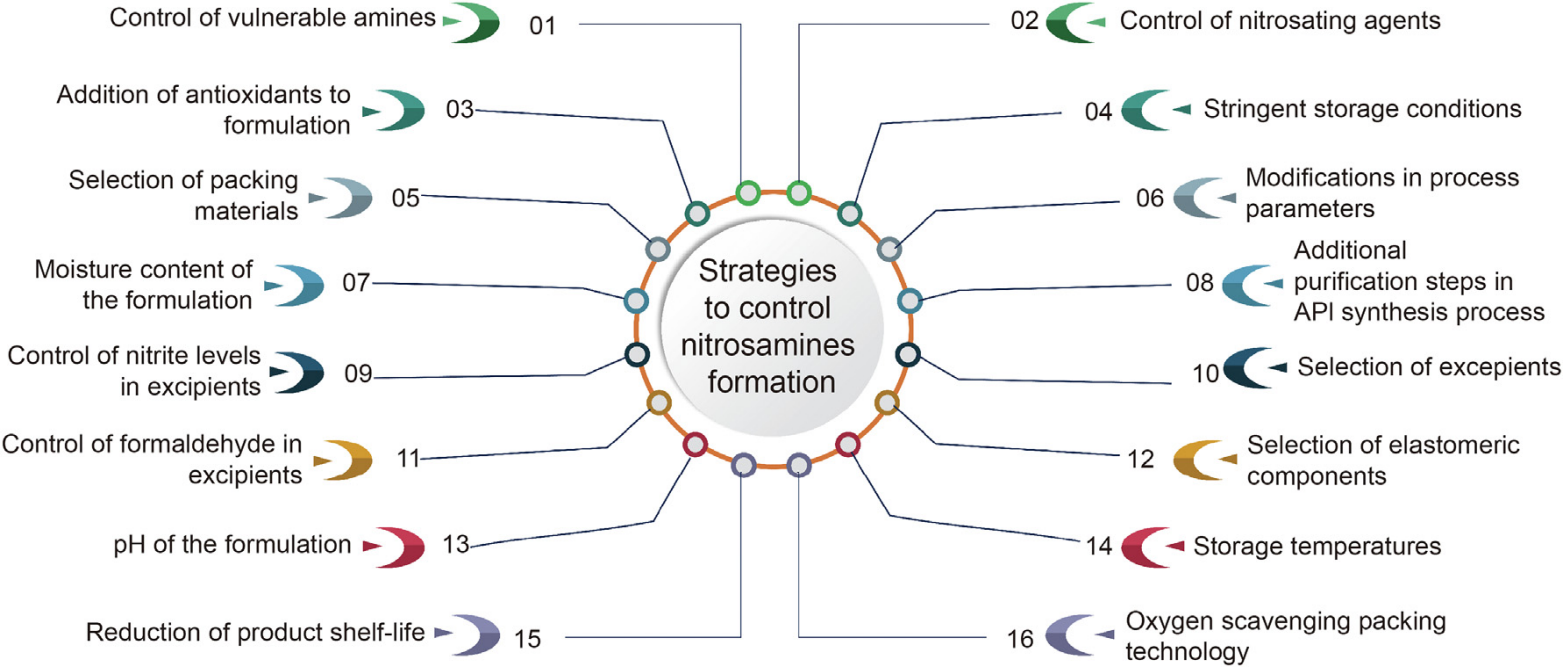
Reformulation strategies to mitigate incorporation of nitrosamine impurities into pharmaceutical products. API: active pharmaceutical ingredient.

During multi-step API synthesis, incorporating additional purification steps can be effective in eliminating nitrosamine formation. It is suggested to avoid reagents containing primary or secondary amine and nitrosating agents and solvents during synthesis process. In assessing the risk of nitrosamine contamination in drug products, various factors, like pH, moisture content, and particle size distribution [185] of the formulation, are to be considered. Material characteristics of drug primary packing materials and excipients used in formulations should also be taken into account. For instance, the choice of blistering material or primer in lidding foil, such as nitrocellulose, can be critical. Nitrocellulose can act as a nitrosating agent when exposed to secondary amines like dimethylamine and diethylamine, which might be present in printing ink. During the blistering process, which is usually done at elevated temperatures, any formed nitrosamine can evaporate and potentially enter into the drug product [186]. Storage conditions may also impact the formation of nitrosamines. For example, the levels of NDMA in ranitidine API and finished products can increase with rising storage temperatures [187]. To mitigate this, Harmon et al. [188] proposed oxygen scavenging packing technology to counter autoxidation, a possible mechanism for the formation of NDMA in ranitidine hydrochloride. Harmon et al. [188] also proposed that formaldehyde, a constituent of various excipients, catalyzes nitrosating reactions and leads to nitrosamine formation. Manufacturers should exercise caution when selecting excipients for their formulations, particularly considering nitrite levels in excipients. This is especially important when drug substance and the drug product contain vulnerable amines in them [189]. Additionally in drug product risk assessment, elastomeric components should also be considered [190]. In context of drug product storage, storage temperatures should be carefully evaluated [117]. Fig. 5 delineates the reformulation strategies to mitigate the incorporation of nitrosamine impurities into pharmaceutical products.

## 7 Conclusion

The systematic review provides a vivid precise risk evaluation of the molecular toxicity pertinent to the nitrosamine impurities, enabling effective root cause analysis and reformulation strategies to mitigate their presence in drug products. The development of highly sensitive analytical methods simplifies the detection process of these impurities. Furthermore, proactive measures by regulatory bodies and research communities are essential in addressing potential nitrosamine impurity-related issues in pharmaceutical products. This approach ensures patient safety and fosters confidence in the recurrent use of medications for various ailments. The presence of *N*-nitroso compounds, particularly *N*-nitrosamines, in drug products, excipients, and APIs poses significant risks to human health due to their genotoxic and mutagenic effects. Emphasis on toxicological data, devising effective methods to calculate acceptable intake limits and exploring structural activity relationships, could significantly help to tackling the crisis of nitrosamines. Sharing knowledge of in-silico predictions, *in vivo* studies and analytical testing on nitrosamines by concerned stakeholders and experts globally can also significantly help in avoid repetitive testing and cost reduction. The review demonstrates the detrimental impact of nitrosamine impurities on physiological systems, emphasizing the importance of novel analytical strategies for sensitive detection and quantification. Additionally, the review highlights the hypothesis that understanding root causes, implementing reformulation strategies, and incorporating regulatory updates can effectively mitigate nitrosamine impurities in pharmaceutical formulations, ensuring the development of safer products.

## CRediT author statement

**Hemanth P.R. Vikram:** Conceptualization, Data curation, Formal analysis, Methodology, Validation, Investigation, Writing - Original draft preparation; **Tegginamath Pramod Kumar:** Conceptualization, Data curation, Formal analysis, Supervision, Funding acquisition, Visualization; **Gunjan Kumar:** Conceptualization, Supervision, Funding acquisition; **Narasimha M. Beeraka** and **Rajashree Deka:** Writing - Reviewing and Editing; **Sheik Mohammed Suhail**, **Sandeep Jat, Namitha Bannimath,** and **Gayatiri Padmanabhan:** Visualization; **Ravandur S. Chandan:** Supervision; **Pramod Kumar:** Conceptualization, Supervision; **Bannimath Gurupadayya:** Supervision, Writing - Reviewing and Editing, Funding acquisition.

## Declaration of competing interest

The authors declare that there are no conflicts of interest.
