# Corrigendum to “Nitrosamines crisis in pharmaceuticals − Insights on toxicological implications, root causes and risk assessment: A systematic review” [J. Pharm. Anal. 14 (2024) 100919]

**DOI:** 10.1016/j.jpha.2024.101033

**Published:** 2024-07-09

**Authors:** Hemanth P.R. Vikram, Tegginamath Pramod Kumar, Gunjan Kumar, Narasimha M. Beeraka, Rajashree Deka, Sheik Mohammed Suhail, Sandeep Jat, Namitha Bannimath, Gayatiri Padmanabhan, Ravandur S. Chandan, Pramod Kumar, Bannimath Gurupadayya

**Affiliations:** a Department of Pharmaceutical Chemistry, JSS College of Pharmacy Mysuru, JSS Academy of Higher Education and Research (JSSAHER), Mysuru, 570015, India; b Xenone Healthcare Pvt. Ltd., New Delhi, 110076, India; c Department of Pharmaceutics, JSS College of Pharmacy Mysuru, JSS Academy of Higher Education and Research (JSSAHER), Mysuru, 570015, India; d Department of Human Anatomy, I.M. Sechenov First Moscow State Medical University (Sechenov University), Moscow, 119991, Russian Federation; e Department of Pharmacology, Raghavendra Institute of Pharmaceutical Education and Research (RIPER), Ananthapuramu, 515721, India; f Herman B. Wells Center for Pediatric Research, Department of Pediatrics, Indiana University School of Medicine, Indianapolis, IN, 46202, USA; g Animal Physiology and Biochemistry Laboratory, Department of Zoology, Gauhati University, Guwahati, 781014, India; h Department of Pharmacology, JSS College of Pharmacy Mysuru, JSS Academy of Higher Education and Research (JSSAHER), Mysuru, 570015, India; i Department of Pharmaceutical Analysis, National Institute of Pharmaceutical Education and Research (NIPER)-Guwahati, Changsari, 781101, India; j Department of Pharmacology, University of Galway, Galway, H91 TK33, Ireland

**Original statement in the Section 2:**

**2. Literature search**

Our primary screening of 180 articles yielded the relevant data for this study.

***2.1. Study selection***

A total of 180 articles spanning from 1960 to the present day, including original research, reviews, case reports and studies reporting nitrosamine impurities above the no-observed-adverse-effect levels (NOAEL) established by regulatory agencies, were initially screened. During the primary screening, we considered factors such as relevance, publication date, access to the full article text, and content. Subsequently, in the secondary screening phase, we excluded the 33 reports including 15 with improper formats (such as case reports, letters, and short communications), 3 with incomplete content, and 15 with unavailable full texts. This process resulted in the selection of 147 articles for further evaluation. Of these, 2 articles were excluded from the study and 2 articles did not describe nitrosamine-induced toxicity or its pharmaceutical or analytical implications. In the end, 143 articles were chosen for systematic review.

**Corrected statement in the Section 2:**

**2. Literature search**

Our primary screening of 190 articles yielded the relevant data for this study.

***2.1. Study selection***

A total of 190 articles spanning from 1960 to the present day, including original research, reviews, case reports and studies reporting nitrosamine impurities above the no-observed-adverse-effect levels (NOAEL) established by regulatory agencies, were initially screened. During the primary screening, we considered factors such as relevance, publication date, access to the full article text, and content. Subsequently, in the secondary screening phase, we excluded the 33 reports including 15 with improper formats (such as case reports, letters, and short communications), 3 with incomplete content, and 15 with unavailable full texts. This process resulted in the selection of 157 articles for further evaluation. Of these, 2 articles were excluded from the study and 2 articles did not describe nitrosamine-induced toxicity or its pharmaceutical or analytical implications. In the end, 153 articles were chosen for systematic review.

The authors regret to find that there is a typographical error in Section 2. The initial and final numbers of articles were misrepresented. The errors did not affect the results and conclusion of the whole study.

**Original Fig. 1:**

**Figure:**
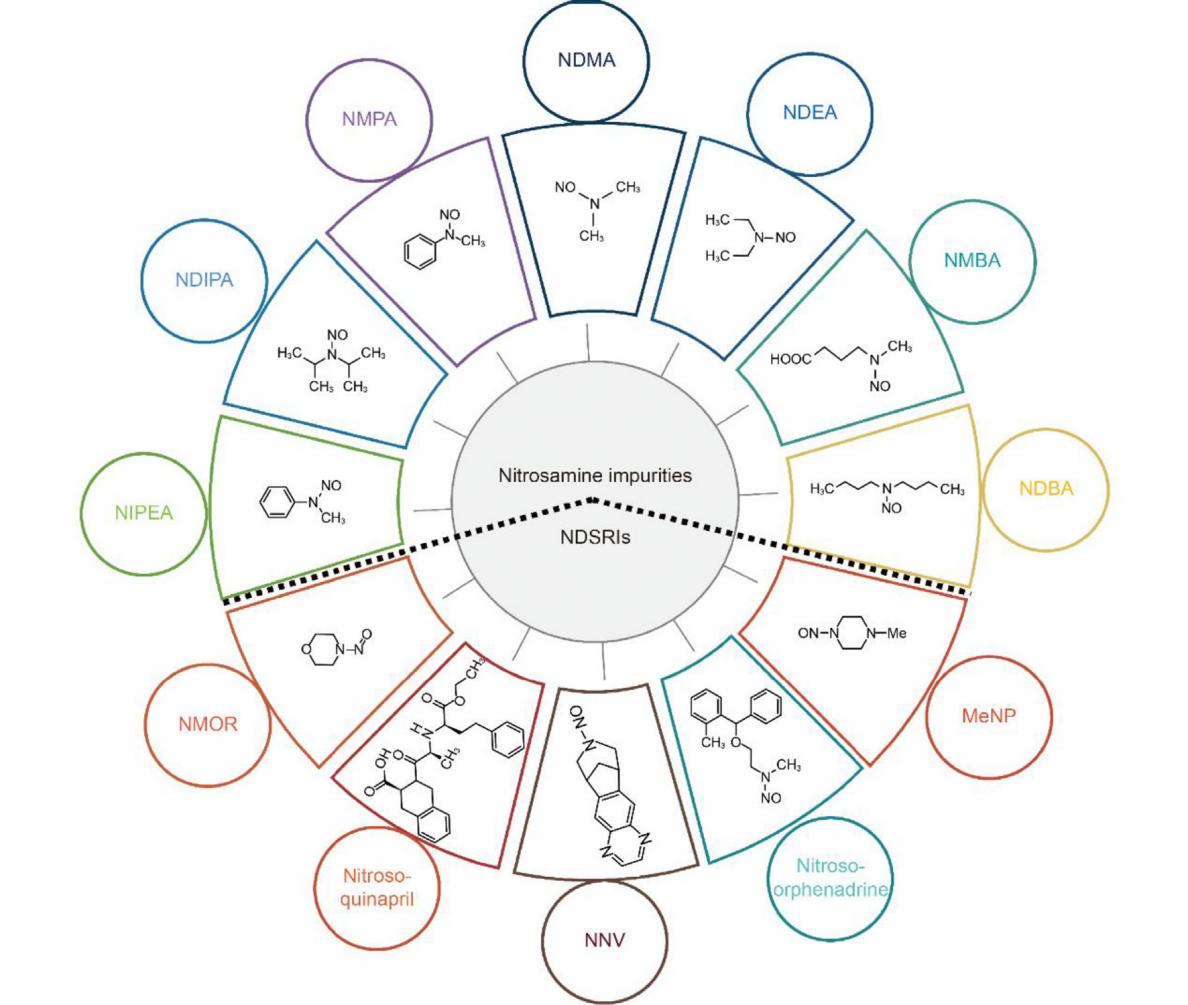

**Corrected Fig. 1:**

**Figure:**
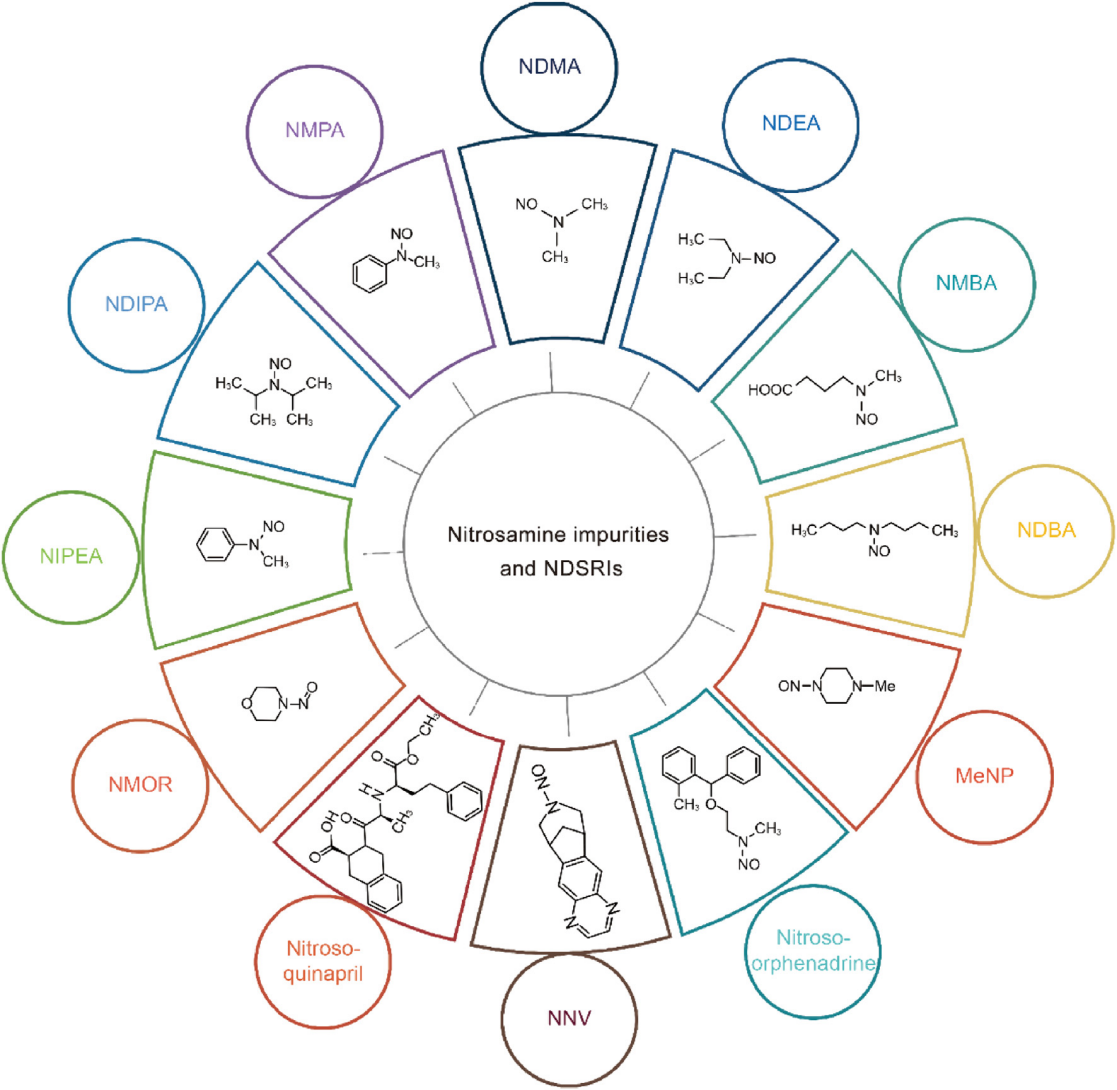

In the originally published Fig. 1, the dotted line is an artifact of the figure template used and does not represent any demarcation within the figure itself. The corrected Fig. 1, with the dotted line removed, avoids confusion for readers. The errors did not affect the results and conclusion of the whole study.

The authors would like to apologize for any inconvenience caused.

