# Supplementary material for: Nitrosamines crisis in pharmaceuticals − Insights on toxicological implications, root causes and risk assessment: A systematic review
Source: J Pharm Anal. 2023 Dec 12;14(5):100919. doi: 10.1016/j.jpha.2023.12.009 (PMC11126534; doi:10.1016/j.jpha.2023.12.009)
Supplement: Multimedia component 1 [file mmc1.docx]

**Table S1**. LC-MS/MS, GC-MS/MS and HPLC methods for nitrosamine analysis.

| S.No | Drug molecule | Nitrosamine  impurity | Analyser | Ionisation source | Matrix | Column | Mobile phase | | | | LOD | LOQ | | Regulatory body/Organization | Refs |
| --- | --- | --- | --- | --- | --- | --- | --- | --- | --- | --- | --- | --- | --- | --- | --- |
| 01 | Losartan-K | NMBA | Q-Trap | APCI | API | Luna^®^ C_8_ (150 mm x 2 mm, 5 µm) | Methanol / Water (65/35) (v/v)- Gradient time program | | | | 8.6 ppb | 28.6 ppb | | EDQM | [139] |
| 02 | Valsartan | NDMA | Triple quadrapole | APCI | Drug product | – | – | | | | 80 ppb | 200 ppb | | EDQM | [140] |
| 03 | Losartan | NDMA  NDEA  NEIPA  NDIPA  NDBA  NMBA | Orbitrap | APCI | API and tablets | Kinetex^®^ F5 100 Å, (100 mm x 4.6 mm, 2.6 µm) | Mobile phase A: 0.1% formic acid in water  Mobile phase B: 0.1% formic acid in methanol | | | | 5 ppb  16 ppb  3 ppb  8 ppb  5 ppb  10 ppb | 50 ppb  50 ppb  50 ppb  50 ppb  50 ppb  50 ppb | | USFDA | [141] |
| 05 | Ranitidine | NDMA | Triple quadrapole | APCI | API and tablets | C_18_-AR, 3 µm 100 Å, (50 mm x 4.6 mm, 3 µm) | Mobile phase A: 0.1% formic acid in water  Mobile phase B: 0.1% formic acid in methanol- Gradient time program | | | | 10 ppb | 33 ppb | | USFDA | [145] |
| 06 | Rifampin and Rifapentine | MNP  CPNP | Orbitrap | ESI | APIs and tablets | Ace Ultracore Super Phenyl Hexyl, 2.5 µm 90 Å, (50 mm x 4.6 mm, 2.5 µm) | Mobile phase A: 10 mM Ammonium Formate in water, pH = 9.0  Mobile phase B: methanol | | | | 10 ppb  3 ppb | 17 ppb  17 ppb | | USFDA | [146] |
| 07 | Metformin | NDMA  NDEA  NEIPA  NDIPA  NDPA  NMPA  NDBA  NMBA | Orbitrap | ESI | API and tablets | Phenomenex Kinetex^®^ Biphenyl 100 Å, (150 mm x 3.0 mm, 2.6 µm ) | Mobile phase A: 0.1% formic acid in water  Mobile phase B: 0.1% formic acid in methanol | | | | 5 ppb  2 ppb  3 ppb  1 ppb  1 ppb  1 ppb  1 ppb  2 ppb | 10 ppb  20 ppb  20 ppb  20 ppb  5 ppb  5 ppb  5 ppb  5 ppb | | USFDA | [147] |
| 08 | Valsartan, Irbesartan, and Losartan | NDMA | Q-trap | APCI | APIs and tablets | – | – | | | | 71 ppb  24 ppb  148 ppb | 236 ppb  79 ppb  492 ppb | | EDQM | [148] |
| 09 | Canagliflozin | NDMA  NEIA  NDIPA  NDEA | – | electron ionization | APIs and tablets | Zorbax SB C18 column (250 x 4.6  mm, 3 µm) | Mobile phase A: 0.1% formic acid in water  Mobile phase B: Acetonitrile  (70:30) | | | | 0.02  0.02  0.015  0.13 | 0.09  0.09  0.09  0.06 | | Mohammed et al. | [149] |
| 11 | Ranitidine | NMBA  NDMA | Q-trap | APCI | APIs and tablets | Phenomenex^®^ Gemini C_18_ (4.6 mm x 100 mm, 3 µm) | Mobile phase: 0.1% formic acid in deionized water  Mobile phase B: 0.1% formic acid in methanol- Gradient time program | | | | 40 ppb  90 ppb | 100 ppb  300 ppb | | HSA | [150] |
| 12 | Ranitidine | NDMA | Triple quadrapole | APCI | APIs and film coated tablets | HSS-T3 (100 mm x 3,0 mm, 1.8 µm), 100 Å | Mobile phase: 0.1% formic acid in deionized water  Mobile phase B: methanol- Gradient time program | | | | 50 ppb | 120 ppb | | EDQM | [151] |
| 13 | Valsartan, Irbesartan, Losartan and Candesartan | AZBT | Q-Trap | APCI | API | Infinity Lab Poroshell 120 PFP, (3.0 mm x 100 mm, 2.7 µm) | Mobile phase: Water LCMS-Grade + 0.1 % Formic acid (LC-MS Grade)  Mobile phase B: Acetonitrile LCMS-Grade/Water LCMS-Grade 95/5 (V/V) + 0.1 % Formic acid (LC-MS Grade)- Gradient time program | | | | 0.4ppm | 0.1ppm | | LGL | [152] |
| 14 | Various sartans | NDMA, NDEA | Orbitrap | APCI | APIs and tablets | – | – | | | |  | 0.1 ppm (all DPs),  0.1 ppm (Valsartan API),  0.2 ppm (other APIs) | | EDQM | [153] |
| 15 | Candesartan Cilexetil, Irebesartan, Losartan, Olmesartan Medoxomil, and Valsartan | NDMA  NDEA  NDELA  NDIPA  NDIPLA  NDPA  NEIPA  NMBA  NMEA  NMOR  NPIP  NPYR | Orbitrap | APCI | APIs | Trifunctional alkyl C_18_ bonded phase, Xselect^®^ HSS T3 column, (150 mm × 3 mm, 3.5 μm) | Mobile phase: 0.1% formic acid in water  Mobile phase B: 0.1% formic acid in acetonitrile/ methanol (2:8)- Gradient time program | | | | 20 ppb  20 ppb  20 ppb  20 ppb  20 ppb  20 ppb  20 ppb  20 ppb  20 ppb  20 ppb  20 ppb  20 ppb | 50 ppb  50 ppb  50 ppb  50 ppb  50 ppb  50 ppb  50 ppb  50 ppb  50 ppb  50 ppb  50 ppb  50 ppb | | - | [154] |
| 16 | Valsartan, Telmisartan, Ranitidine, Nizatidine, and Metformin | NDMA | Orbitrap | APCI | APIs | Shimpack ARATA C_18_, (75 mm × 30 mm, 2.2 μm) | Mobile phase: water:acetonitrile:formic acid (990:10:1)  Mobile phase B: water:acetonitrile:formic acid (100:900:1)- Gradient time program | | | | – | 30 ppb | | Yamamoto et al. | [155] |
| 17 | Telmisartan | NDMA  NDEA  NEIPA  NMBA  NDIPA  NDBA | Triple quadrapole | APCI | API |  |  | | | | 2 ppb  2 ppb  2 ppb  2 ppb  2 ppb  2 ppb | 4 ppb  4 ppb  4 ppb  4 ppb  4 ppb  4 ppb | |  | [156] |
| 18 | Losartan and hydrochlorothiazide | NDMA  NDBA  NDEA  NDIPA  NEIPA  NMBA  NMEA | Triple quadrapole | APCI | tablets | Agilent Pursuit XRs Ultra diphenyl column (150 × 2.0 mm, 2.8 μm) | Mobile phase: 0.1% formic acid in water  Mobile phase B: | | | | 0.25–0.5 ng/mL | 0.25–0.5 ng/mL | | R. Patel et al. | [157] |
| 19 | Metformin along with Glibenclamide, Gliclazide, Glipizide, Glimepiride Evogliptin, | Eight Nitrosamine Impurities | Triple quadrupole | APCI | Drug product | Phenomenex Kinetex^®^ (150 mm x 3.0 mm) | **Mobile phase A:** 0.1% formic acid **Mobile phase B:** 0.1% formic acid in methanol- Gradient time program | | | | - | - | | R. Solanki et al. | [158] |
| GC-MS/MS METHODS | | | | | | | | | | | | | | | |
| Sl.No | Drug molecule | Nitrosamine  impurity | Analyzer | Ionization source | Matrix | column | | LOD | LOQ | | | | Regulatory body/Organization | | Refs |
| 01 | Valsartan, Losartan, Irbesartan, Olmesartan, Candesartan | NDMA, NDEA, EIPNA, DIPNA, DPNA, DBNA | Triple quadrapole | Electron Ionization | APIs and tablets | Column 1 (Inlet to EPC): VF-624ms, 30m, 0.25 mm ID, 1.4 µm Film  Column 2 (EPC to MS): Deactivated Fused Silica, 1.35m, 0.15 mm ID. | | N/A | 15 ppb | | | | EDQM | | [159] |
| 02 | Dichloromet-hane,  Ethyl- Acetate, Toluene,  O-xylene | NDMA, NDEA, NEIPA, NDIPA, NDPA, and NDBA | Triple quadrapole | electron ionization |  | VF-WAXms (30 m x 0.25 mm I.D, coated with 1.00 µm film) | | 13 ppb, 13 ppb, 13 ppb, 13 ppb, 13 ppb and 4.3 ppb | 5 ppb, 5 ppb, 5 ppb, 5 ppb, 5 ppb and 13 ppb | | | | E.R. Kosuri et al. | | [160] |
| 03 | All sartan | NDMA | Single Quadrupole | Electron Ionization | APIs and tablets | Restek Rtx-624 (30 mm x 0.25 mm, 1.4µm) | | 40 ppb | – | | | | EDQM | | [161] |
| 04 | All sartan | NDMA  NDEA | Triple quadrapole | Electron Ionization | APIs and tablets | – | | 2.0 ppb  2.0 ppb | 5.4 ppb  7.3 ppb | | | | Health Canada | | [162] |
| 05 | Various sartans, metformin and ranitidine. | NDMA,  NDEA | Triple quadrapole | Electron Ionization | APIs and tablets | DB-624 (60 m x 0.25 mm id x1.4 μm) | | 0.3 μg/kg  0.07 μg/kg | 0.9 μg/kg  0.3 μg/kg | | | | H.-H Lim et al. | | [124] |
| 07 | Valsartan | NDMA | Single Quadrupole | Electron Ionization | APIs and tablets | DB-Wax GC Column, (30 m x 0.25 mm, 0.5 µm) | | 50 ppb  20 ppb | 300 ppb  50 ppb | | | | US FDA | | [163] |
| 08 | Valsartan | NDMA  NDEA  NEIPA  NDIPA  NDBA | Triple quadrapole | Electron Ionization | APIs and tablets | VF-WAXms GC Column, (30 m x 0.25 mm, 1.00 µm) | | 5 ppb  1 ppb  1 ppb  1 ppb  10 ppb | 8 ppb  5 ppb  5 ppb  5 ppb  25 ppb | | | | US FDA | | [164] |
| 09 | Valsartan | NDMA, NDEA, NDIPA, NEIPA | Triple quadrapole | Electron Ionization | APIs and tablets | DB-Wax GC Column, (30 m x 0.25 mm, 0.5 µm) | | 10 ppb  10 ppb  25 ppb  50 ppb | 50 ppb  50 ppb  50 ppb  50 ppb | | | | US FDA | | [165] |
| 10 | Metformin | NDMA | Orbitrap | GC–HRMS (Orbitrap) | Drug formulation | HP – INNOWAX (30m x 0.25mm x 0.25μm) | | 10 ppb | 30 ppb | | | | HSA | | [166] |
| 11 | Ranitidine | NDMA | Orbitrap | Electron Ionization | APIs and tablets | DB-Wax (30 m × 0.25 mm , 0.5 μm) | | 23.3 ppb | 116.7 ppb | | | | Y.M. Alshehri et.al | | [121] |
| 12 | Sunitinib malate Olmesartan medoxomil  Cilostazol. | NDMA, NMEA,  NDEA, NDBA, NMOR, NPYR, NPIP, NDPA, and N-methyl-npz | Single quadruple | Electron ionization | APIs | VF-WAXms (30 m × 0.32 mm, film thickness of 1.0 µm) | | 0.15 to 1.00 ng/mL | 0.15 to 1.00 ng/mL | | | | A. B. Witkowska et.al | | [167] |
| **HPLC METHODS** | | | | | | | | | | | | | | | |
| **Sl.No** | **Nitrosamine impurity** | | **Matrix** | **Detector** | **Column** | **Mobile phase** | | **LOD** | | **LOQ** | | | | **Reference** | |
| 01 | NDMA  NMEA  NDEA  NDPA  NDBA  NPIP  NPYR | | Meat | UV ( 231 nm ) | C_18_ (250 mm × 4.6 mm, 5 μm) | 10 mM ammonium hydroxide pH = 8.9 and acetonitrile | | 0.48 µg/mL  0.61 µg/mL  0.33 µg/mL  0.46 µg/mL  0.30 µg/mL  0.53 µg/mL | | 1.616 µg/mL  2.029 µg/mL  1.097 µg/mL  1.550 µg/mL  1.011 µg/mL  1.775 µg/mL | | | | [168] | |
| 02 | NEDLA  NDMA  NDEA  NSC336  NDPA  NSC134  NPIP  NDBA | | Food product ( salted duck eggs) | UV ( 233 nm ) | Hypersil GOLD C_18_ column | Mobile phase A: water  Mobile phase B: acetonitrile  Gradient method | | 0.13 µg/kg  0.19 µg/kg  0.09 µg/kg  0.08 µg/kg  0.11 µg/kg  0.28 µg/kg  0.14 µg/kg  0.55 µg/kg | | 0.42 µg/kg  0.64 µg/kg  0.30 µg/kg  0.26 µg/kg  0.37 µg/kg  0.92 µg/kg  0.45 µg/kg  1.82 µg/kg | | | | [169] | |
| 03 | NDMA | | water samples | Fluorescence | NovaPak^®^ C_18_ column (3.9 mm × 150 mm, 4µm) | Acetonitrile–water mixture (2:1, in volume) | | – | | – | | | | [170] | |
| 04 | NMOR  NDMA  NDEA  NPYR  NPIP | | Food products | Fluorescence | C_18_ column | acetonitrile–water (55:45, v/v) | | 8 pg  27 pg  75 pg  11 pg  34 pg | | – | | | | [139] | |
| 05 | NPYR  NDMA  NDEA  NDPA  NDBA | | Food products | Fluorescence | ZORBAX SB-C_18_ (4.6 mm×150 mm, 5 μm) | Mobile phase A: 5% acetonitrile in water  Mobile phase B: acetonitrile | | 0.06 ng/g  0.03 ng/g  0.04 ng/g  0.01 ng/g  0.07 ng/g | | 0.19 ng/g  0.11 ng/g  0.15 ng/g  0.03 ng/g  0.21 ng/g | | | | [171] | |
| 06 | NDMA  NDEA | | Enalapril maleate | Fluorescence | NovaPak C_18_, (150 mm × 3.9 mm) 4 µm | Mobile phase: water  Mobile phase B: acetonitrile | | 0.013 μg/g  0.017 μg/g | | 0.038 μg/g  0.050 μg/g | | | | [172] | |
| 07 | NDMA | | Valsartan- API and tablets | Fluorescence | Unison UK-C_18_ column (250 mm× 4.6 mm, 3 μm) | water-acetonitrile mobile phase containing 0.1% formic acid  gradient elution | | 0.0085 μg/mL | | 0.0285 μg/mL | | | | [173] | |
| 08 | NDMA  NDEA  NMOR  NPIP  NYPR | | Water | UV | ODS column | acetonitrile–water containing 5 mM acetate buffer at pH 4.0 | | – | | – | | | | [174] | |
| 09 | NDMA  NMBA  NDEA  NEIPA  NDIPA  NDBA | | Valsartan- API and tablets | UV | C_18_ (250 × 4.6 mm, 5 μm) | Acetonitrile, methanol and water (pH 3.2) – Gradient time program | | 0.013 μg/mL  0.011 μg/mL  0.006 μg/mL  0.011 μg/mL 0.007 μg/mL 0.011 μg/mL | | 0.041 μg/mL  0.034 μg/mL  0.020 μg/mL  0.035 μg/mL  0.022 μg/mL 0.034 μg/mL | | | | [175] | |

LC-MS/MS: Liquid Chromatography with tandem mass spectrometry; Q-Trap: Quadropule ion trap; APCI: Atmospheric pressure chemical ionization; LOD: Limit of Detection; LOQ: Limit of Quantitation; ppb: Part per billion; ppm: parts per million; NMBA: N-nitroso-N-methyl-4-aminobutanoic acid; NDMA: N-Nitrosodimethylamine; EDQM: European Directorate for the Quality of Medicines & Healthcare; NDEA: N-nitrosodiethylamine; NEIPA: N-nitrosoisopropylethyl amine; NDIPA: N-nitrosodiisopropylamine; USFDA: United States Food and Drug Administration; MNP: 1-methyl-4-nitrosopiperazine; CPNP: 1-cyclopentyl-4-nitrosopiperazine; NDPA: N-Nitrosodipropylamine; NMPA: N-nitrosomethylphenylamine; HAS: Health Science Authority Singapore, AZBT: azidomethyl-biphenyl-tetrazole; NDELA: N-Nitrosodiethanolamine; NDIPA: N-nitrosodiisopropylamine; NMEA: N-nitrosomethylethylamine; NPYR: N-nitrosopiperidine; NMOR: N-nitrosomorpholine; NPIP: N-nitrosopiperidine; GC-MS/MS: Gas Chromatography Tandem Mass Spectrometry; EIPNA: N-nitrosoethylisopropylamine; DIPNA: N-nitrosodiisopropylamine; DPNA: diisopropyl-N-nitrosamine.
